# Supplementary material for: Acceptance and compliance with micronutrient powder (MNP) among children aged 6–23 months in northern Nigeria
Source: PLOS Glob Public Health. 2022 Oct 17;2(10):e0000961. doi: 10.1371/journal.pgph.0000961 (PMC10022258; doi:10.1371/journal.pgph.0000961)
Supplement: S1 Dataset — (ZIP) [file pgph.0000961.s008.zip › S8_Dataset/Spot Checks.zip!/Spot Checks/05072015_A_SO_PM_2_SC b.pdf]

SPOT CHECK RECORDING FORM

State: Adamawa **LGA: ____Song____ Community: _Wurokoncha_**

Name of caregiver (female): Aminatu Bulus

Name or caregiver (male): ………………………

Sex of index child: _female___ Age of index child (in months): _ 10 months________

Spot Check Information

1. **Date of MNP distribution to household** (circle option below based on LGA name above)**:**
   - **Kebbi:** BirninKebbi&Jega LGA: **June 16**
   - **Kebbi:** Danko-wasagu: **June 17**
   - **Adamawa:**Song, Ganye: **(June 18)**
   - **Adamawa:**Guyuk **June 19**
2. **Date of Spot Check Observation: ____5/7/2015_____**
3. **Number of sachets remaining**(Ask the caregiver to show you and count the specific number of sachets remaining)**:**

   42

4. **Number of sachets that should remainusing one MNP sachet per day** (Analyst only)**:**

   44

5. **Number of sachets remaining / number of sachets should be remaining**(Analyst only)**:**

_____42____ / ___44______ = __95.5__ %

Probes

Question 1. You have **XX** MNP sachets remaining. Explain how easy or difficult it was to use the MNP product on a daily basis for your child 6 – 23 months? (Circle one answer)

(Very Difficult) **Difficult** **Easy** **Very Easy**

Question 2. Describe any specific challenges that you faced that made the MNP difficult to use on a daily basis?

- I sometimes forget but have to be reminded by my husband
- The baby has been sick for some time now
- I don't know the exact importance of the powder

Question 3. Describe specific aspects of the MNP product that you or your child enjoyed that made it easier to use on a daily basis?

- None. For now because I need more clarification and explanation about this powder.

Question 4. What questions do you have about the MNP based on your experience using it?

- Is this powder a drug or what?
- Is it only in kunu that can be use to mix it.
- What of if I forget to give the baby for say two days. What is the implication.

Question 5. What specific recommendations do you have that we can do as a program to make the MNP easier to use on a daily basis?

I can't think of any for now
